# Tuning the Sensing Properties of N and S Co-Doped Carbon Dots for Colorimetric Detection of Copper and Cobalt in Water

**DOI:** 10.3390/s22072487

**Published:** 2022-03-24

**Authors:** Ramanand Bisauriya, Simonetta Antonaroli, Matteo Ardini, Francesco Angelucci, Antonella Ricci, Roberto Pizzoferrato

**Affiliations:** 1 Department of Industrial Engineering, University of Rome Tor Vergata, 00133 Rome, Italy; ramanand.bisauriya@students.uniroma2.eu; 2 Department of Chemical Sciences and Technology, University of Rome Tor Vergata, 00133 Rome, Italy; simonetta.antonaroli@uniroma2.it; 3 Department of Life, Health and Environmental Sciences, University of L’Aquila, 67100 L’Aquila, Italy; matteo.ardini@univaq.it (M.A.); francesco.angelucci@univaq.it (F.A.); 4 Faculty of Bioscience and Technologies for Food, Agriculture and Environment, University of Teramo, 64100 Teramo, Italy; aricci@unite.it

**Keywords:** optical sensing, water quality, divalent copper, colorimetry, N and S co-doped carbon dots

## Abstract

In this study, nitrogen and sulfur co-doped carbon dots (NS-CDs) were investigated for the detection of heavy metals in water through absorption-based colorimetric response. NS-CDs were synthesized by a simple one-pot hydrothermal method and characterized by TEM, STEM-coupled with energy dispersive X-ray analysis, NMR, and IR spectroscopy. Addition of Cu(II) ions to NS-CD aqueous solutions gave origin to a distinct absorption band at 660 nm which was attributed to the formation of cuprammonium complexes through coordination with amino functional groups of NS-CDs. Absorbance increased linearly with Cu(II) concentration in the range 1–100 µM and enabled a limit of detection of 200 nM. No response was observed with the other tested metals, including Fe(III) which, however, appreciably decreased sensitivity to copper. Increase of pH of the NS-CD solution up to 9.5 greatly reduced this interference effect and enhanced the response to Cu(II), thus confirming the different nature of the two interactions. In addition, a concurrent response to Co(II) appeared in a different spectral region, thus suggesting the possibility of dual-species multiple sensitivity. The present method neither requires any other reagents nor any previous assay treatment and thus can be a promising candidate for low-cost monitoring of copper onsite and by unskilled personnel.

## 1. Introduction

Copper belongs to a relatively small group of heavy metals (HMs) that are essential to life and human health by playing a key role in many biochemical and physiological processes. In fact, copper is necessary for metabolic functions and is critical in several proteins and metalloenzymes, such as superoxide dismutase. It contributes to the formation of red blood cells and helps to preserve the health of nerve cells and the immune system. Copper also acts as an important antioxidant, thus reducing the damage produced by free radicals to DNA [1,2,3]. On the other hand, high doses of copper produce general environmental pollution and can be very dangerous for human health. For instance, excessive concentration of copper ions in drinking water, i.e., in the order of milligrams per liter, can cause various diseases such as liver and kidney damage, oxidative stress, and Parkinson’s and Alzheimer’s diseases [4,5,6]. Hence, it is highly desirable to develop efficient and reliable detection methods to evaluate the presence of copper in the environment and in biological systems. In particular, quantification of Cu(II) in water has recently attracted increasing attention because of the growing, widespread contamination of surface waters coming from worldwide use of copper in several industrial sectors, especially electrochemistry, electrical wires, machine parts, fertilizers, and batteries [7].

Accurate and reliable determination of Cu(II) is usually carried out through established analytical techniques including, but not limited to, inductively coupled plasma mass spectrometry (ICP-MS), high performance liquid chromatography (HPLC), atomic absorption spectrometry (AAS), atomic fluorescence spectroscopy (AFS), and graphite furnace atomic absorption spectroscopy (GFAAS) [8,9,10,11,12,13]. However, these laboratory methods, which ensure trace-level sensitivity, need sophisticated, bulky, heavy, and expensive instrumentation. In addition, the necessary assay procedures are generally complex, time consuming, and require highly skilled staff [14,15]. For this reason, alternative strategies for simpler, portable, and faster detection are in high demand in view of a diffuse and massive onsite monitoring, even at the expense of a lower sensitivity. Fluorescence and absorption spectroscopy have recently been investigated as detection methods that can provide easier procedures and a great potentiality due to the continuously broadening range of possible sensing materials. These two optical techniques, based on light emission and variations of absorbance, respectively, offer different and, in some cases, complementary advantages and drawbacks. Fluorimetric methods generally show higher sensitivity and dynamic range, taking advantage of the null background, and greater versatility, due to the chance of using either turn-off or turn-on effects [16]. However, they require more advanced instrumentation, i.e., light source and revelator, while fluorescent materials are often based on complex synthesis steps [17,18]. On the other hand, variations of absorbance can enable simple naked-eye visual inspection through visual color change along with instrumental quantitative colorimetric determination of the presence of a specific analyte [19,20,21].

Recently, fluorescence-based sensing has extensively been investigated in carbon dots (CDs), also referred to as graphene quantum dots (GQDs), a class of carbon nanoparticles that have attracted considerable attention because of the peculiar characteristics and plentiful preparation methods, including “bottom-up” and “top-down” approaches. Development of simple one-pot methods enabled easy synthesis of doped CDs and significantly enhanced their properties, such as tunable chemical activity together with robust chemical inertness, low cytotoxicity, excellent biocompatibility, low photobleaching with stable photoluminescence (PL) properties [22,23], and novel applications [24]. Even though a certain variety of structures can be produced, CDs are generally considered as anisotropic nanoparticles with lateral dimensions in the range 2–10 nm and much smaller thickness. They are made of isolated sp^2^-graphitic carbon domains surrounded by a highly defective sp^3^ carbon framework. Importantly, all the synthesis strategies, including strong acid oxidization, pyrolysis, high-temperature hydrothermal methods, etc., necessarily introduce a significant number of oxygen-containing moieties, such as carboxyl groups, mainly attached at the edges, and hydroxyl, carbonyl, and epoxy groups located on the basal surface of nanosheets [25]. These functional groups make CDs hydrophilic, thus enabling stable water dispersions, and are deemed to play a significant role in fluorescent emission. Heteroatom doping, especially with nitrogen (N) and more recently with sulfur (S) and their combination, is also frequently performed, achieving a noticeable increase of the quantum yield of fluorescence emission and its tuning to longer wavelengths [26]. It is because the N and S atoms can produce additional electron pairs, new defects sites, and active sites (due to their comparable atomic size in the case of C and N (the radius of C: 0.77 Å, N: 0.75 Å, S: 1.02 Å), strong valence bonds, and similar electronegativity in the case of C and S (electronegativity of C: 2.55, S: 2.58, N: 3.04)), which would significantly modify the local chemical activities of the carbon nanostructures. Specifically, these active sites can induce electron transfer between the heavy metal ions and the doped CDs. Finally, some residues of the starting compounds can stick to surface of CDs through electrostatic or Van der Waals interaction and produce additional sensing properties, as will be discussed for o-Phenylenediamine in the present case.

In fact, a great deal of research has focused on the synthesis of fluorescent CDs for chelating with and selectively detecting different heavy metal ions [27,28,29]. Within this framework, fluorescent sensing of Cu(II) ions has been the topic of many studies, both exploiting fluorescence quenching and turn-on effects [30,31,32,33,34,35]. Differently, absorption-based sensing of copper through CDs has rarely been reported [36] and deserves further investigations in view of applications to easy-to-use visual colorimetric devices.

In this paper, we reported on the synthesis of water solutions of N, S co-doped CDs (NS-CDs) by a simple one-pot hydrothermal method and using o-Phenylenediamine as the precursor along with the ammonium sulfate as the source of N and S. NS-CDs detected the presence of Cu(II) metal ions in aqueous solution directly and sensitively with a colorimetric method by simple addition of the contaminated water sample, without further treatment. The sensing mechanism of NS-CDs was investigated using UV–Vis spectroscopy in the presence of various metal ions, and high sensitivity and selectivity to Cu(II) were observed over a wide class of other metal ions, with a linearity range from 1 to 100 µM and with a detection limit of 100 nM.

## 2. Materials and Methods

### 2.1. Materials

O-phenylenediamine and ammonium sulphate were used as carbon, and N and S sources, respectively. The following heavy metal salts were used for the sensitivity and selectivity tests: Hg_2_(NO_3_)_2_⋅2H_2_O, CrCl_3_⋅6H_2_O, Pb(NO_3_)_2_, Fe(NO_3_)_3_⋅9H_2_O, CuCl_2_⋅2H_2_O, Cd(NO_3_)_2_⋅4H_2_O, CoCl_2_⋅6H_2_O, NiCl_2_⋅6H_2_O, and NaAsO_2_ and AgNO_3_. All chemicals were purchased from Merck Sigma Aldrich (Merk Life Science S.r.l., Milano, Italy) at analytical reagents (AR) grade and used as received without any purification. All solutions were prepared using Milli-Q de-ionized water ((18.25 MΩ cm, Millipore, Milford, MA, USA), DI). Stock solutions of each metal at a concentration of 10 mmol/L were prepared in DI water in advanced and diluted to appropriate concentration just before the experiment.

### 2.2. Synthesis of N and S Co-Doped CDs

To synthesize the NS-CDs, two separate solutions of o-Phenylenediamine (0.4 M) and ammonium sulfate (0.4 M) were prepared in 12 mL of absolute ethanol and 12 mL of DI-water, respectively. The two solutions were mixed and stirred for 20 min. The mixed solution was then transferred to a Teflon-lined (100 mL) stainless sealed autoclave and heated at 220 °C for 6 h. After the reaction, the autoclave was cooled down to room temperature naturally. The product, which was dark brown, was filtered through a 0.22 µm polyethersulphone membrane and dialyzed against DI water through a dialysis bag with a molecular weight cut-off of 2 kDa (Sigma Aldrich—Merk Life Science S.r.l., Milano, Italy) for 24 h to remove the excess precursor. It was then dissolved with DI water (V_NS-CDs_/V_DI_: 1/60) to obtain what is therein referred to as NS-CDs sensing solution. This solution was characterized in detail and the pH was varied with nitric acid and sodium hydroxide for pH studies.

### 2.3. Instrumentation for Characterization and Sensitivity Measurements

Fourier transform infrared spectra (FT-IR) were acquired in the range between 400 and 4000 cm^−1^ using a Perkin Elmer Spectrum 100 FT-IR spectrometer (PerkinElmer Italia Spa, Milano, Italy). The NS-CD aqueous solutions were dried for 24 h at 50 °C in nitrogen atmosphere and the solid residues were analyzed in KBr cells. All the measurements were performed at room temperature unless a specified otherwise. NMR spectra were obtained by drying 10 mL of NS-CD solution at 40 °C for 20 h. Once the water was removed from the sample, the solid product was diluted in 0.6 mL of dimethylsulphoxided-6 (99.96 atom % D. Sigma Aldrich - Merk Life Science S.r.l., Milano, Italy) and transferred into a 5 mm NMR tube. The studies were performed with Varian 700 MHz equipment (Varian Inc., Palo Alto, CA, USA).

The characterization of the NS-CDs size and homogeneity before and after interaction with metal ions was carried out by bright-field transmission electron microscopy (TEM) at room temperature using a CM100 electron microscope (Philips Electron Optics, Eindhoven, Netherlands) equipped with high-contrast objective lens, a lanthanum hexaboride (LaB_6_) filament, and a CMOS Phurona camera (EMSIS GmbH, Münster, Germany). The micrographs were acquired at 46,000–245,000× magnification while applying an electron energy of 100 keV. The specimens were made as described herein. First, the sample solutions were prepared by mixing 25 μL of 20 μM NS-CDs and 25 μL of 200 μM, 1 mM, or 2 mM CuSO_4_ and allowed to incubate 30 min at room temperature under stirring. After incubation, the solutions were centrifuged 10 min at room temperature and 10,000 rpm before removing the supernatant volumes and resuspending the pellets in equal volumes of DI water to remove any unbound NS-CDs and metal ions. The washing step was carried out three times and the final samples were used to prepare the grids for TEM analysis as follows. First, a drop of 20 μL DI water was placed on a flat piece of parafilm; 5 μL of sample solutions were placed onto the carbon side of 300 mesh gold grids coated with ultrathin carbon film (3 nm thickness) on lacey carbon support (Ted Pella, Inc., Redding, CA, USA), gripped upwards using negative locking tweezers and let to adsorb 1 min before blotting off the sample volume by touching the grids edge with filter paper (Whatman); the grids were then washed by touching the carbon-coated side with the water drop followed by blotting off with filter paper (Whatman, Maidston, UK). Finally, the grids were air-dried 30 min at room temperature with the carbon-coated side facing up before TEM imaging.

The elemental analysis of the NS-CDs after interaction with metal ions was carried out at room temperature by scanning electron microscopy (SEM) using a Gemini SEM 500 (Carl Zeiss Microscopy GmbH, Jena, Germany) with an annular detector aSTEM and a spectroscope for energy dispersion spectroscopy (EDS) equipped with a Ultim Max 100 detector (Oxford Instruments NanoAnalysis, High Wycombe, UK). The specimens were the same as those used for TEM analysis (see above). The images, EDS spectra, and maps were acquired at 296,000× magnification and applying an electron energy of 20 kV.

UV–Vis absorption spectra were recorded with a Cary 50 spectrophotometer (Varian Inc., Palo Alto, CA, USA). Photoluminescence measurements were carried out by using a laboratory setup based on a 200 W continuous discharge Hg(Xe) lamp (Oriel Instruments, Stratford, CT, USA) as the excitation light source, followed by a 25-cm monochromator (Photon Technology International, Inc., Birmingham, NJ, USA). PL light was dispersed by a 25-cm monochromator (Oriel Cornerstone 260, Oriel Instruments, Stratford, CT, USA) and revealed by a Hamamatsu R3896 photomultiplier (Hamamatsu Photonics Italia Srl., Milano, Italy). Optical-path fused silica cuvettes of 10 mm were adopted for the liquid samples both in the UV–Vis absorption and PL.

### 2.4. Determination of Copper(II) Ions and Interference Studies

All the measurements for Cu(II) ion determination were performed against a reference (blank) solution that was prepared by adding 1 mL of DI water to 1 mL of NS-CDs sensing solution and gently stirring the mixture for 10 s. The typical experiment in the presence of heavy metal (HM) ions was performed by adding 1 mL of the specific HM salt solution in DI water at the prescribed ion concentration to 1 mL of NS-CDs sensing solution and gently stirring for 10 s. For sensing in real water, the same procedure as for DI was adopted with the only difference being that the 1 mL real water samples were spiked with 10 µL of highly concentrated salt solutions in DI water to achieve the prescribed HM assay concentration. Before spiking, real water samples were filtered through a 0.22 µm filter without any further treatment. All the measurements were performed in triplicate. All the spectra were recorded 10 minutes after mixing the water samples with the NS-CD sensing solution.

## 3. Results and Discussion

### 3.1. Morphological, Structural, and Optical Characterization of NS-CDs

**Figure 1:**
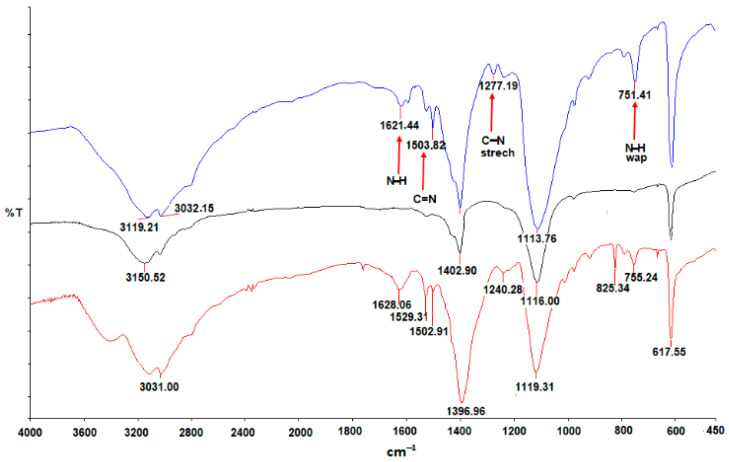
FTIR spectra of the dried samples: as-prepared NS-CDs sensing solution (blue curve), NS-CDs + Cu(II) (black), and NS-CDs + Fe(III) (red).

The sensing solution was characterized by FT-IR spectroscopy to determine the type of functional groups added to the carbon structure during the synthesis. Furthermore, the morphology and average size of NS-CDs were estimated by TEM imaging. The optical properties of CDs were investigated by means of absorbance as well as fluorescence spectroscopies in the UV–Vis range. Figure 1 displays the Fourier transform infrared spectra (FTIR) recorded for the dried (40 °C for 2 h under nitrogen flux) reference NS-CDs sample before (blue curve) and after the interaction with Cu(II) (black) and Fe(III) ions (red). In the spectrum of pristine NS-CDs, some peaks are pointed out by arrows and attributed to the presence of different typical nitrogen functional groups produced by the hydrothermal synthesis of N-doped CDs [25,37]. Peaks at 1113 cm^−1^ and 1400 cm^−1^ may come from S=O bond and C-S bond, respectively, and indicate the presence of sulfur in NS-CDs [36]. Noticeable differences can be observed in the spectrum of NS-CDs + Cu(II) with all the peaks indicated by arrows strongly reduced due to interaction with Cu(II). This suggests that copper ions coordinate with CDs mainly through amino groups to form Cu–CD complexes, as will be discussed in Section 3.2. On the other hand, addition of Fe(III) did not modify the peaks at 1621 cm^−1^ and 751 cm^−1^ appreciably, thus suggesting a weak interaction of this ion with N groups, while the emergence of a peak at 825 cm^−1^ indicates coordination through phenolic groups.

**Figure 2:**
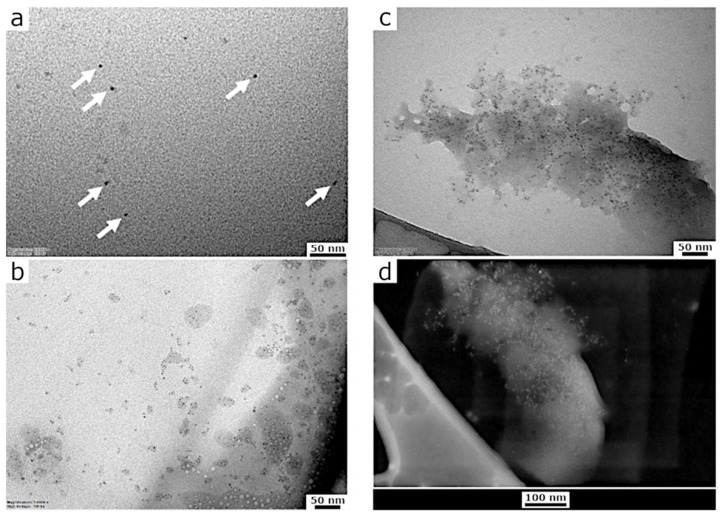
Micrographs of NS-CDs. TEM of single NS-CDs (**a**) or small clusters (**b**) observed before the interaction with Cu(II) ions. The white arrows highlight the single NS-CDs showing a diameter of approximately 5 nm. Large clusters are observed upon interaction with the metal ions (**c**). STEM micrograph of the same area confirming the presence of NS-CDs clusters after interaction (**d**).

Figure 2a reports a TEM micrograph of the reference NS-CDs sample after adsorption onto an ultrathin carbon layer on gold grids. The NS-CDs appear mainly as dark monodispersed particles with dimeters of 5 nm, approximately. Rare but present, some NS-CDs form small clusters likely due to an artifact of the drying step of the sample preparation as shown in Figure 2b. Figure 2c shows the effect of the interaction between NS-CDs and Cu(II) upon incubation and washing to remove unbound metal ions. The micrographs display large clusters of aggregated particles. The effect of the interaction is clearly visible even in solution as the NS-CDs precipitate into a dark material which can be harvested upon centrifugation; conversely, the reference Cu(II)-free sample remains colloidal (data not shown).

**Figure 3:**
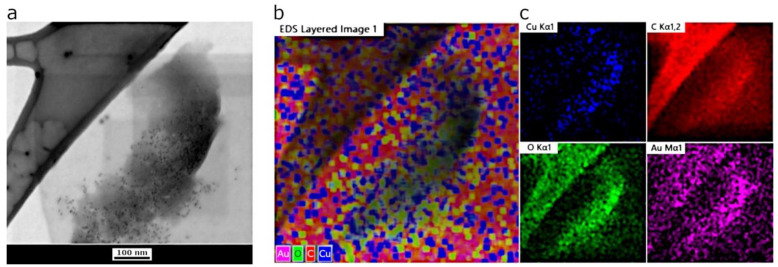
EDS chemical analysis of NS-CDs. TEM of NS-CDs clusters after interaction with Cu(II) ions (**a**); and related EDS maps (**b**,**c**). The elemental analysis shows the presence of the ions indicating that Cu(II) was complexed with the NS-CDs. Other elements such as carbon and oxygen were detected along the NS-CDs clusters likely due to the carbon layer of the grid where the sample was deposited. Also note the presence of other metals such as gold likely due to the gold grid supporting the carbon layer (see Section 2).

Furthermore, additional analysis was carried out to characterize the elemental content of the sample after interaction with Cu(II) by means of STEM and EDS microanalysis. Figure 3a displays a TEM micrograph collected using the SEM microscope upon interaction with metal ions (same area as shown in Figure 2c,d). The chemical characterization was carried out in this area by detecting the EDS signals of the most abundant elements as shown in Figure 3b. The image showed the EDS maps with clearly visible signals from copper onto the NS-CDs cluster, which becomes much less evident in the empty area of the grid outside the cluster indicating that Cu(II) is complexed with the NS-CDs as expected. Other representative elements have been found alongside copper such as carbon and oxygen as well as gold, the latter likely due to the metal composition of the support (see Section 2).

**Figure 4:**
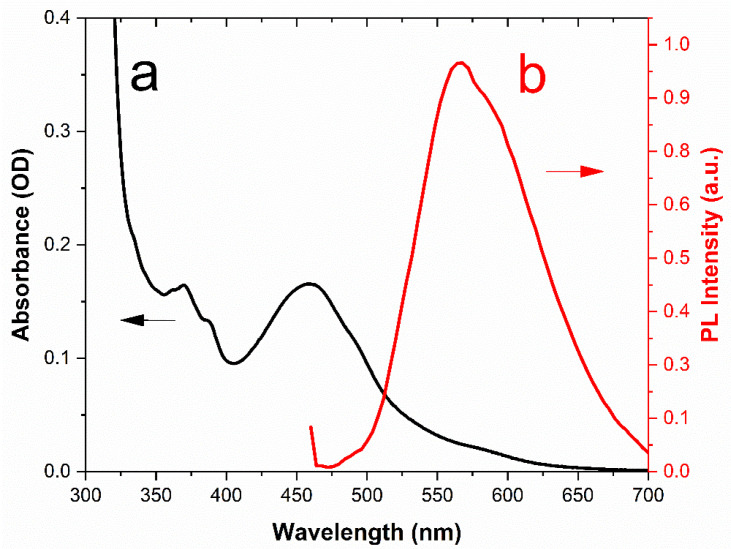
UV–Vis absorption (a); and fluorescence (b) spectrum of NS-CDs sensing solution.

Figure 4 displays the UV–Vis absorption spectrum (black line) and the PL spectrum after excitation at λ_exc_ = 430 nm (red line) of NS-CDs sensing solution. The steep increase of absorbance towards the short-wavelength side is generally attributed to the tail of the high-energy π-π* optical transitions occurring in isolated residual sp^2^-carbon domains in the highly defective graphene-like carbon lattice [37]. Similarly, the n–π* transitions of nitrogen or oxygen containing functional groups introduced by doping into the NS-CDs, as revealed by the FTIR analysis, can explain the small peaks that emerge from the tail in the range 360–380 nm [38]. Differently, the distinct absorption band peaked at 460 nm originates from the presence of residual oxidized o-Phenylenediamine (OPD_ox_) that is adsorbed on the surface of CDs through electrostatic interaction [35,39], as will be discussed in Section 3.2. This is also confirmed by the photoluminescence (PL) spectrum after excitation at 430 nm (red line) that lies in the typical emission range of OPD_ox_.

### 3.2. Sensing Response towards Cu(II) Ions

The sensing properties of NS-CDs were investigated against the presence of various common or heavy metal ions, i.e., Cu(II), Fe(III), Pb(II), Cd(II), Ag(I), Cr(VI), Ni(II), Co(II), As(III), and Hg(II). For quantification and understanding of selectivity and color change, UV–Vis absorption spectra of NS-CDs sensing solution were recorded over the whole visible range after the addition of water samples with the different metal ions at a concentration of 100 µM. Initially, this investigation was carried out by using the as-prepared NS-CDs sensing solution, as described in Section 2.2, which showed a pH of 8.3.

**Figure 5:**
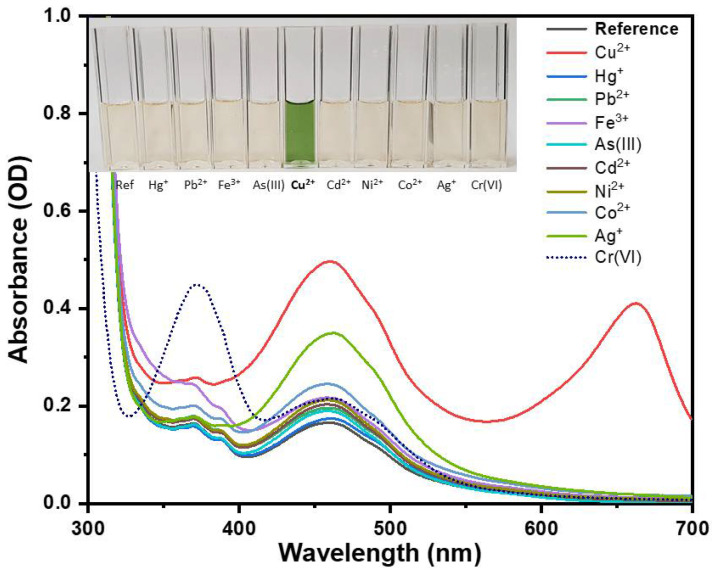
Selective response of UV–Vis absorption spectra of as-prepared NS-CDs sensing solution upon the addition of different HM ions at a concentration of 100 µM. Note that As(III) is present as the anion AsO_3_^3−^ in water while Cr(VI) is present as CrO_4_^2−^ and Cr_2_O_7_^2−^, which is in contrast to all the other metals, which are present as cations.

Figure 5 shows that the interaction of NS-CDs with Cu(II) produced an appreciable increase of absorption over all the visible range and, more importantly, the appearance of a distinct peak at 660 nm, approximately. From the point of view of the perceived color, the emergence of the band at 660 nm turned the sensing solution from light yellow to green, as displayed in the inset of Figure 5. Differently, the other metal ions did not give origin to significant changes in the profile of absorbance except for a minor general increase below 550 nm. It should be noted that Ag(I) ions also caused a significant enhancement of the band at 460 nm which will be discussed below. Finally, it should be noted that the peak at 360 nm in the presence of Cr(VI) is simply due to the absorbance of chromium ions themselves. All these variations, however, did not alter the perceived color appreciably (see the inset of Figure 5) and the NS-CDs showed high selectivity to Cu(II).

The physical origin of the emergence of the peak at 660 nm can be attributed to the Cu(II) ions binding to the CDs through the N-containing functional groups. This leads to the formation of cuprammonium complexes that exhibit absorption bands in the region from 630 to 700 nm [40,41]. Such a mechanism of chelation of copper by the amino groups is consistent with the strong reduction of the respective signals observed in FTIR spectra, as discussed above (see Figure 1). On the other hand, the increase of the band at 460 nm can be assigned to the oxidizing action of copper towards OPD in the presence of CDs, as it was investigated for sensing in OPD-based systems [39,42]. This effect is also visible in the comparison of the ^1^H NMR spectra of NS-CDs and NS-CDs + Cu(II) reported in Figure S1 of Supplementary Information. The differences between 6 and 8 ppm, in the range of H aromatic signals, highlight the presence of oxidized OPD in the compound coordinated with the Cu(II). Moreover, the signals between 1 and 1.6 ppm, possibly due to the presence of H from linear alkanes of NS-CDs, are significantly modified. Interestingly, oxidation was also observed to occur in the presence of Ag(I) [42], which explains the significant enhancement of the band at 460 nm that we recorded with this metal as well (see Figure 5). It should be noted that a similar formation of metal-nanoparticle complexes, in the presence of different HMs, is commonly observed both in CDs prepared with different top-down methods [43] and in fluorescent metallic nanoparticles [44].

Figure S2, which displays the UV–Vis absorption spectra of water solutions of bare HM ions at a concentration of 100 µM, clearly shows that the absorbance by pure HM ions does not contribute appreciably to the variations observed after the interaction with NS-CDs. Moreover, Figure S3 shows that the perceived color of the sensing solution presents slight but appreciable changes even for concentrations below the guideline value set by the WHO.

**Figure 6:**
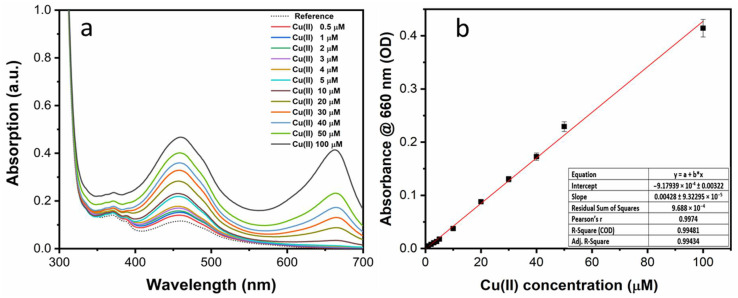
(**a**) UV–Vis absorption spectra of the as-prepared NS-CDs sensing solution upon the addition of Cu(II) ions at different concentrations; (**b**) calibration curve.

For a quantitative determination, spectrophotometric titration was performed upon incremental addition of Cu(II) ions. With the increase of ion concentration, both the absorption peak at 660 nm and the band at 460 nm increased gradually as shown in Figure 6a. Specifically, the relationship between the value of absorbance at 660 nm and the Cu(II) concentration (calibration curve) showed a linear trend in the range 1–100 µM (Figure 6b).

As reported in Figure S4, the linear trend is substantially confirmed in the low-concentration range. This enabled the estimate of the limit of detection (LOD) of the present method for Cu^2+^ according to the given Equation (1)

LOD = 3 σ/m(1)

where σ is the standard deviation (n = 3) of the reference absorbance data and m is the slope of the linear calibration curve. The calculated LOD for Cu(II) was 200 nM, which is much lower than the permissible limits set by either the WHO ~30 μM (2 mg/L) or EPA ~20 μM (1.3 mg/L) in drinking water [45]. In addition, Figure S5 shows that the pH conditions of the water samples had little effect on the response to Cu(II) in the range from 4 to 8.

### 3.3. Interfering Effects from Other HMs and Optimization of the Sensing Solution

**Figure 7:**
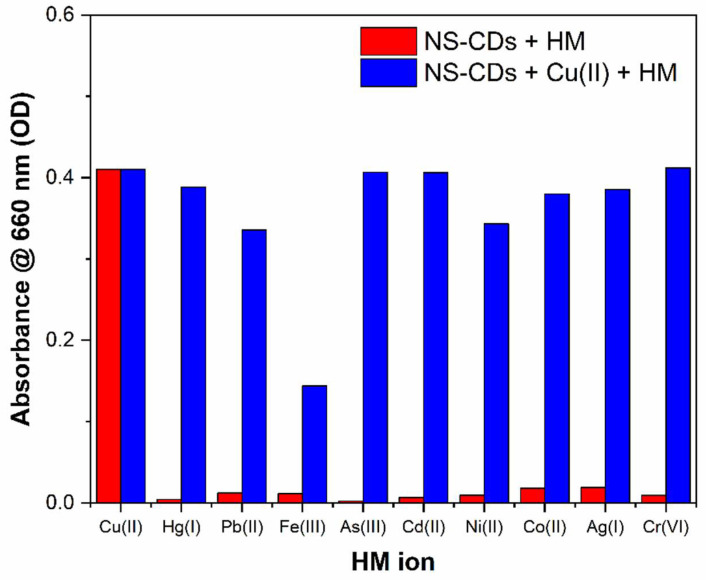
Selective response (red bars) and interference from other HMs (blue bars) in the as-prepared NS-CD sensing solution.

The use of NS-CDs as a colorimetric sensor for Cu(II) was also tested with the other different metal cations for the competing selectivity and interference effects. In this experiment, water samples containing both copper and the specific metal cations were used, with each species at a concentration of 100 μM. As shown in the absorption spectra of Figure S6 and summarized in the diagram of Figure 7 (blue bars), Fe(III) showed a significant masking effect on the detection of Cu(II) by reducing the response to 40% of its initial value. This effect is not surprising if one considers that most of the reported nitrogen-doped CDs presented a great affinity to iron, generally attributed to the special coordination interaction between Fe(III) and the phenolic hydroxyl groups on the surface of CDs [46]. In the present case, Fe(III) ions, even without altering the optical absorption and the FTIR spectra, could compete with and prevent binding of Cu(II). Therefore, inspired by the different nature of the two interactions and by recent results indicating the pH-dependency of Fe(III) coordination [47], we tried to optimize selectivity and reduce masking effects by changing the pH of the NS-CDs sensing solution. Specifically, since we wanted to exclude the interference from buffer solutions, as well as noninherent groups, we adjusted pH by adding only nitric acid and sodium hydroxide, as shown in Figure 7.

The effect of pH of the sensing solution on the masking effect of Fe(III) towards Cu(II) can be seen in the spectra of Figure S7a and in the graph of Figure S7b. As observed, the interference from iron decreased while the sensitivity to copper increased as the pH shifted towards more basic conditions. This behavior can be attributed to the fact that different metal hydroxides form under diverse pH and concentration conditions [47]. In the present case, for a pH of 9.5, the response to Cu(II) was decreased only by 18% in the presence of Fe(III), which is quite an acceptable effect. Therefore, we repeated the sensing experiments with this pH-optimized sensing solution, as reported in the following paragraphs.

### 3.4. Optical Response of the pH-Optimized Sensing Solution

**Figure 8:**
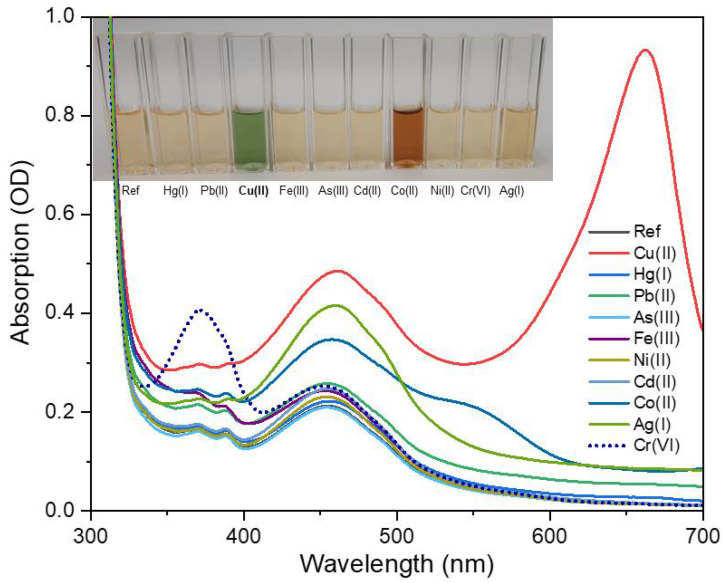
Selective response of UV–Vis absorption spectra of pH-optimized NS-CDs sensing solution upon the addition of different HM ions at a concentration of 100 µM. Note that As(III) is present as the anion AsO_3_^3−^ in water while Cr(VI) is present as CrO_4_^2−^ and Cr_2_O_7_^2−^, which is in contrast to all the other metals, which are present as cations.

As shown in Figure 8, which should be compared with Figure 5, the optical response to Cu(II) of the pH-optimized sensing solution was clearly higher than that of the as-prepared solution, with the peak at 660 nm doubling its intensity and significantly exceeding the band at 460 nm. The selectivity remained good even though, surprisingly, the solution also appreciably responded to Co(II) through the increase of absorbance in the range from 400 to 600 nm and a peculiar shoulder at 550 nm. Like in the case of copper, these variations could be attributed to the formation of amminecobaltate complexes through binding to the N-containing functional groups [48]. The inset of Figure 5 shows that the spectral change resulted in a clear color change from light yellow to orange/brown. Within the framework of visual observation, the pH-optimized sensing solution behaved as a dual-sensitive detector. Importantly, as reported in Figure S8, no significant changes could be observed in the UV–Vis absorption and PL spectra of pure NS-CDs upon the variation of the pH.

**Figure 9:**
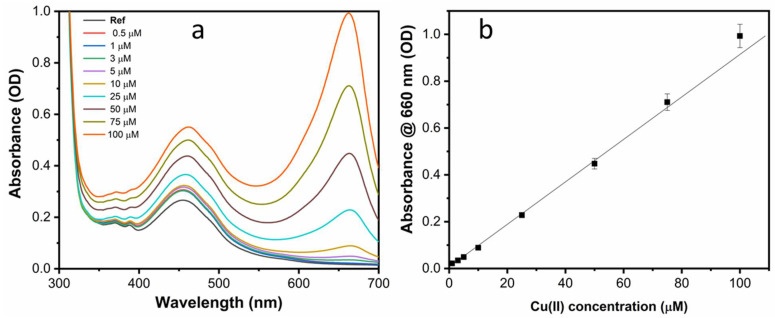
(**a**) UV–Vis absorption spectra of the pH-optimized NS-CDs sensing solution upon the addition of Cu(II) ions at different concentrations; (**b**) calibration curve.

The titration test is reported in Figure 9a and showed that the intensity of the peak at 660 nm increased linearly with the Cu(II) concentration with a two-fold increase of sensitivity in comparison to that of as-prepared sensing solution (see Figure 9b). The LOD of 100 nM for Cu(II) in water could thus be estimated.

**Figure 10:**
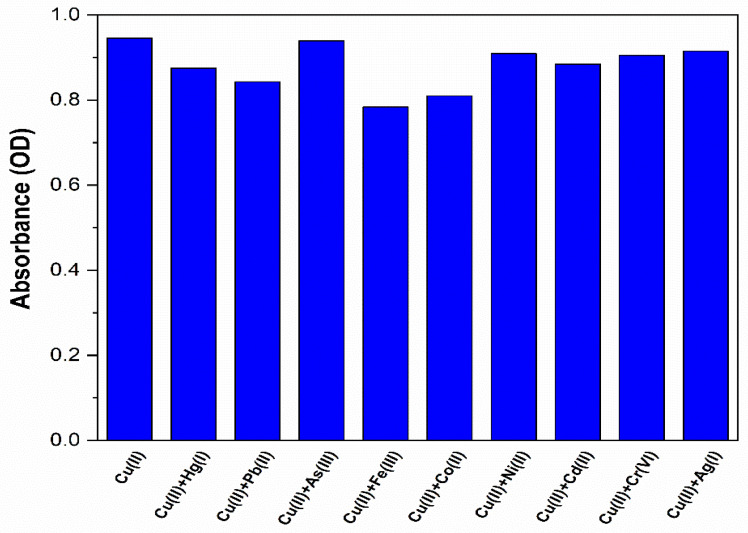
Interference from other HMs (blue bars) in the pH-optimized NS-CDs sensing solution.

Despite the additional sensitivity to Co(II), the masking effects were generally quite small, as shown in the diagram of Figure 10 (blue bars), since the interference from cobalt ions lies in a different spectral range in comparison to the absorption band produced by Cu(II) (Figure S9). In particular, as anticipated in Section 3.3, the interference by Fe(III) was reduced to 18%.

The different optical response of pH-optimized NS-CD sensing solution was paralleled by the different behavior of FTIR spectra (see Figure S10 vs. Figure 1). Specifically, the peak at 1120 cm^−1^ was appreciably reduced in pH-optimized NS-CDs and in NS-CDs + Cu(II) while it completely disappeared after addition of Fe(III), indicating that iron strongly interacted with the sulfur groups instead of coordinating with phenolic hydroxyls. On the other hand, no appreciable variations were observed after addition of either Cu(II) or Fe(III) in the signals at 825 cm^−1^, 1503 cm^−1^, 1529 cm^−1^, and 3420 cm^−1^, suggesting that both N and phenolic hydroxyl groups play a minor role at high pH.

### 3.5. Sensing Experiments in Real Water Samples

To investigate the practical applicability of this method, we carried out standard sensing measurements on spiked samples of tap water of the city water mains of Rome (south-east area) and of lake water collected from Lago di Castel Gandolfo, a small volcanic lake near Rome. Specifically, 1 mL of a real water sample was simply filtered through a 0.2 μm filter and spiked with 10 μL of DI solution at appropriate Cu(II) concentrations. The sample was then added to 1 mL of pH-optimized sensing solution without any further treatment such as centrifugation, acid digestion, or boiling. Table 1 shows that the percentage recovery of the spiked samples for the lake water was in the range from 95% to 102%, which represents a good result, especially when considering that the water samples simply underwent mechanical filtration. In the tap water samples, recovery varied from 96% to 104% in the high concentration range and worsened to 92% for the lowest measured value of 5 µM. Considering the high permanent hardness of the drinking water in Rome (33°fH), with a calcium content as high as 101 mg/L (2.5 mM), these results are also reasonably compatible with the real practicability of this method. Moreover, Figure S11 shows that the calibration curve obtained in the DI water is essentially reproduced by sensing in real water, which is not often reported in the literature.

### 3.6. Comparison with Other Sensing Materials and Techniques

Table 2 shows the detection limit and linearity range of other techniques and nanomaterials that have recently been reported for detection of Cu(II) in comparison with the present system. The LOD of the present method (0.1 µM) is outperformed by that of other nanomaterial systems, e.g., Au nanoclusters [49], but it is still much lower than the guideline value set by the WHO (30 µM). On the other hand, the linearity range is broader than that of all of the other methods considered; the exception is the method described by Kim and collaborators [50] which, however, presented a significantly higher LOD (2 µM) and was based on the Cu(II)-assisted oxidative coupling of silica nanoparticle-supported aniline with 4-aminoantipyrine to form a chromogenic quinonediimine dye. In fact, most of the reported colorimetric methods rely on quite complex and time-consuming sensing procedures. For instance, the fluorescence detection of copper with OPD-derived carbon dots [35] required that the stock solutions of the metal ions were first diluted with 10 mM of phosphate buffered saline (PBS) at pH 7.0 prior to use and then mixed with10 mM PBS (pH 7.0) containing C dots and Cu(II) ions for incubation at 37 °C for 1 h. Similarly, the impressive LOD of 0.1 nM obtained with gold nanoparticles [49] was based on the peroxidase-like activity of histidine-Au nanoclusters (His-AuNCs) and required mixing 36 μL of 25.0 mM 3,3′,5,5′-tetramethylbenzidine (TMB), 150 μL of the His-AuNCs stock solution, 30 μL of solution, and 90 μL of 10 M H_2_O_2_ into 2.694 mL of 0.2 M pH 3.0 acetate buffer at 25 °C. Differently, the present sensing experiment only required the addition of the water sample to the sensing solution in a 1:1 volume ratio, and the result could be obtained in 10 min at room temperature without the needs of pH buffer solutions or any other procedure.

## 4. Conclusions

In summary, by using a simple one-pot hydrothermal method, we synthesized stable aqueous solutions of nitrogen and sulfur co-doped carbon dots (NS-CDs) that showed a peculiar selective colorimetric response to the presence of Cu(II) ions in water through variation of the optical absorption and perceived color. This sensing procedure only required the addition of the water sample to the sensing solution in a 1:1 ratio and a reaction time of 10 min without the needs of any other step. FTIR, STEM, and NMR analyses confirmed that the absorption band at 660 nm, appearing upon interaction with Cu(II), could be assigned to coordination of copper with NS-CDs through the N-containing functional groups and formation of cuprammonium complexes. NS-CDs also played a minor role in catalyzing the oxidation of OPD by copper. The absorbance at 660 nm was linear with the copper concentration in the broad interval of 1–100 µM and enabled a limit of detection of 200 nM, which is well below the guideline value set by the WHO (30 µM). Response to copper was highly selective against other most common HMs. However, the sensitivity was significatively reduced by the simultaneous presence of Fe(III) ions, likely due to the high affinity of iron towards the phenolic hydroxyl groups of NS-CDs. This hypothesis was confirmed by the fact that the interference effects of Fe(III) were effectively lowered by increasing the pH of the sensing solution up to 9.5, while the sensitivity to Cu(II) was doubled and enabled an improved LOD of 100 nM. In such relatively strong basic conditions, the sensing solution also responded to the presence of Co(II) with a distinct absorbance band at 550 nm which, however, did not interfere with the sensitivity to Cu(II). In fact, by using appropriate spectral analysis, this behavior could allow multiple selective sensitivity with a single sensing material. We think that this tuning procedure could be of inspiration for investigations on other CDs and that the NS-CDs are a promising material for the user-friendly, simple, and onsite monitoring of Cu(II) in water, possibly by incorporation of NS-CDs into solid transparent matrices for more practical visual sensing systems.

## Figures and Tables

**Table 1:** The recovery of Cu(II) ions in real lake and tap water samples (n = 3).

Sample	Spiked Cu(II) (µM)	Measured Cu(II) (µM)	Recovery (%) *	RSD (%)
Lake water Lago di Castel Gandolfo (Italy)	50	51	102	2.1
	25	25.1	100.5	1.7
	5	4.75	95	2.6
Tap water (Rome, Italy)	50	52	104	1.9
	25	24	96	1.8
	5	4.6	92	2.8

* Measured/Spiked (%).

**Table 2:** Comparison of the performances of various sensing techniques and nanomaterials for Cu(II).

Materials	Method	Linear Range (µM)	Limit of Detection (µM)	Reference
Au NCs	Colorimetric	0.001–0.1	0.0001	[49]
Si NPs	Colorimetric	3–200	2	[50]
Dibenzo[b,j][1,10]Phenanthroline	Colorimetric	10–100	0.14	[51]
Semiconductor QDs	Colorimetric	0.025–2.5	0.01	[52]
Receptor L	Colorimetric	0–50	2.82	[53]
Rhodamine	Colorimetric	0–30	0.48	[54]
CDs	Colorimetric Fluorescence	0.01–10 0.1–2	0.004 0.09	[55]
CDs	Fluorescence	0.5–7	0.15	[8]
N-CDs	Fluorescence	0.05–25	0.023	[56]
BPEI-UCNPs	Fluorescence	0.05–10	0.04	[57]
Si NPs	Fluorescence	0.05–10	0.03	[58]
Adenine-stabilized CDs	Fluorescence	0.001–0.75	0.0003	[59]
CDs	Fluorescence	0.002–0.080	0.0018	[35]
NS-CDs	Colorimetric	1–100	0.1	Present study

## Data Availability

Not applicable.

## Supplementary Materials

The following supporting information can be downloaded at: https://www.mdpi.com/article/10.3390/s22072487/s1, Figure S1: Comparison of the 1H NMR spectra of (a) NS-CDs and (b) NS-CDs + Cu(II); Figure S2: UV-vis absorption spectra of water solutions of HM ions at a concentration of 100 µM; Figure S3: Color variations of the as-prepared NS-CDs sensing solution upon the addition of Cu(II) ions in the low range of concentrations; Figure S4: UV-vis absorption spectra (left) and calibration curve (right) of the as-prepared NS-CDs sensing solution upon the addition of Cu(II) ions in the low range of concentrations; Figure S5: Dependance of absorbance at 660 nm of as-prepared NS-CDs sensing solution on the pH of the DI water samples with 100 µM of Cu(II); Figure S6: UV-vis absorption spectra of as-prepared NS-CDs sensing solution upon the addition of Cu(II) and other interfering HM ions at a concentration of 100 µMm; Figure S7: (a) UV-vis absorption spectra of NS-CDs sensing solution upon the addition of Cu(II) and Fe(III) at a concentration of 100 µM for different values of pH of sensing solution; (b) Absorbance at 660 nm with Cu(II) in the presence of Fe(III) (blue circles) and in absence (red squares) for different values of pH of sensing solution; Figure S8: Comparison of UV-vis absorption spectra (left) and fluorescence spectra (right) of as-prepared NS-CDs sensing solution (red curves) and pH-adjusted sensing solution (black); Figure S9: UV-vis absorption spectra of pH-optimized NS-CD sensing solution upon the addition of Cu(II) and other interfering HM ions at a concentration of 100 µM; Figure S10: FTIR spectra of the dried samples: pH-optimized NS-CDs sensing solution (blue curve), NS-CDs + Cu(II) (black) and NS-CDs + Fe(III) (red); Figure S11: Measurements in real water samples in comparison with the calibration curve obtained in DI water.
