# Supplementary material for: Tuning the Sensing Properties of N and S Co-Doped Carbon Dots for Colorimetric Detection of Copper and Cobalt in Water
Source: Sensors (Basel). 2022 Mar 24;22(7):2487. doi: 10.3390/s22072487 (PMC9003535; doi:10.3390/s22072487)
Supplement: Supplementary file 1 [file sensors-22-02487-s001.zip › sensors-1642715-supplementary.pdf]

sensors

MDPI

Supplementary Material

Tuning the Sensing Properties of N and S Co-Doped Carbon Dots for Colorimetric Detection of Copper and Cobalt in Water

Ramanand Bisauriya [1], Simonetta Antonaroli [2], Matteo Ardini [3], Francesco Angelucci [3], Antonella Ricci [4] and Roberto Pizzoferrato [1,*]

[1] Department of Industrial Engineering, University of Rome Tor Vergata, 00133 Rome, Italy; ramanand.bisauriya@students.uniroma2.eu
[2] Department of Chemical Sciences and Technology, University of Rome Tor Vergata, 00133 Rome, Italy; simonetta.antonaroli@uniroma2.it
[3] Department of Life, Health and Environmental Sciences, University of L'Aquila, 67100 L'Aquila, Italy; matteo.ardini@univaq.it (M.A.); francesco.angelucci@univaq.it (F.A.)
[4] Faculty of Bioscience and Technologies for Food, Agriculture and Environment, University of Teramo, 64100 Teramo, Italy; aricci@unite.it
* Correspondence: pizzoferrato@uniroma2.it

Citation: Bisauriya, R.; Antonaroli, S.; Ardini, M.; Angelucci, F.; Ricci, A.; Pizzoferrato, R. Tuning the Sensing Properties of N and S Co-Doped Carbon Dots for Colorimetric Detection of Copper and Cobalt in Water. *Sensors* **2022**, *22*, 2487. https://doi.org/10.3390/s22072487

Academic Editor: Cecilia Cristea

Received: 2 March 2022
Accepted: 22 March 2022
Published: 24 March 2022

Publisher's Note: MDPI stays neutral with regard to jurisdictional claims in published maps and institutional affiliations.

Copyright: © 2022 by the authors. Licensee MDPI, Basel, Switzerland. This article is an open access article distributed under the terms and conditions of the Creative Commons Attribution (CC BY) license (https://creativecommons.org/licenses/by/4.0/).

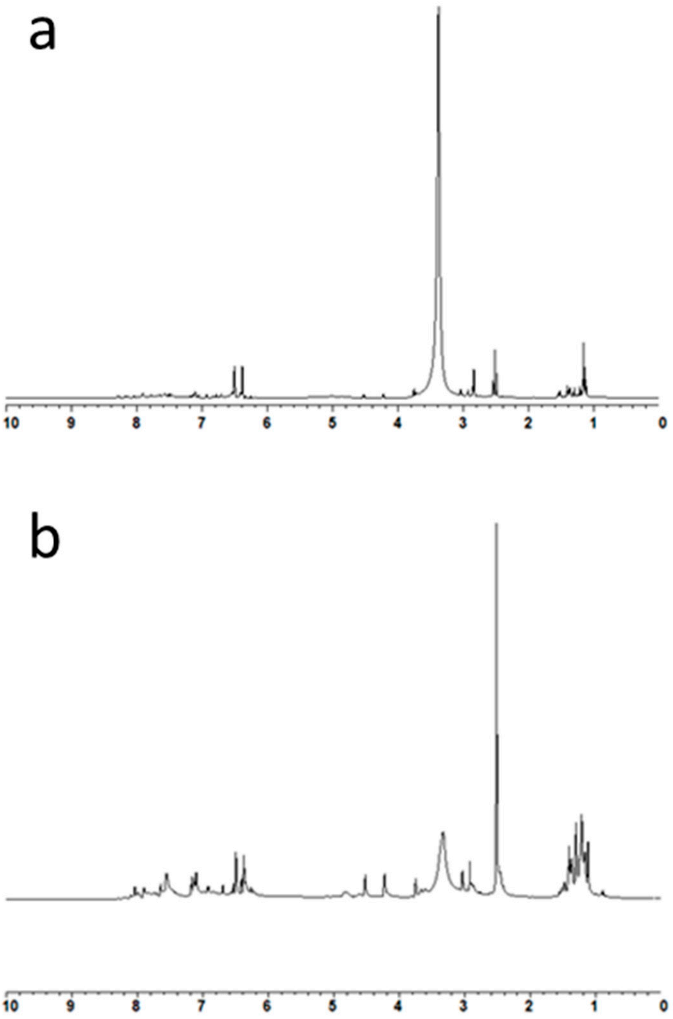

Figure S1. Comparison of the 1H NMR spectra of (a) NS-CDs and (b) NS-CDs + Cu(II).

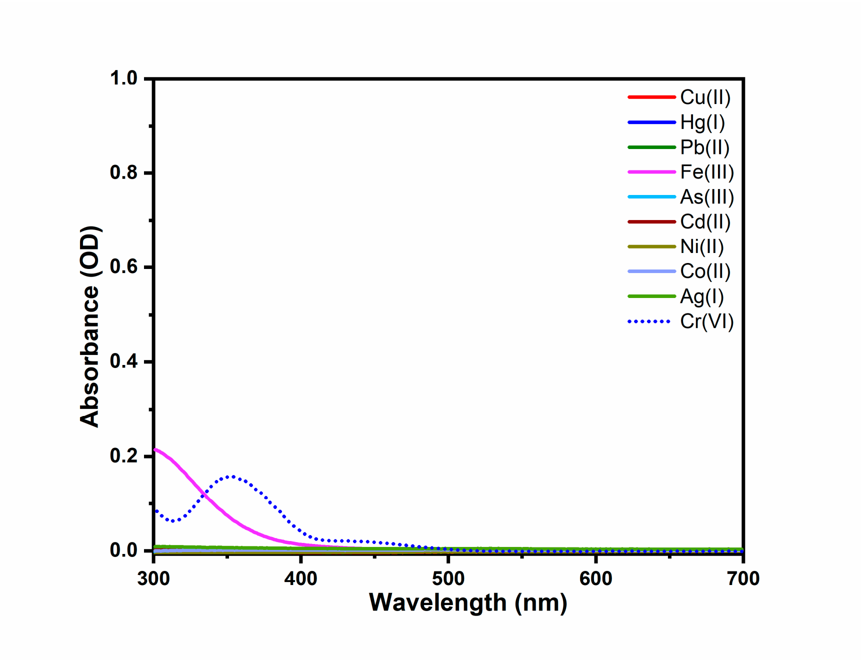

Figure S2. UV-vis absorption spectra of water solutions of HM ions at a concentration of 100 μM.

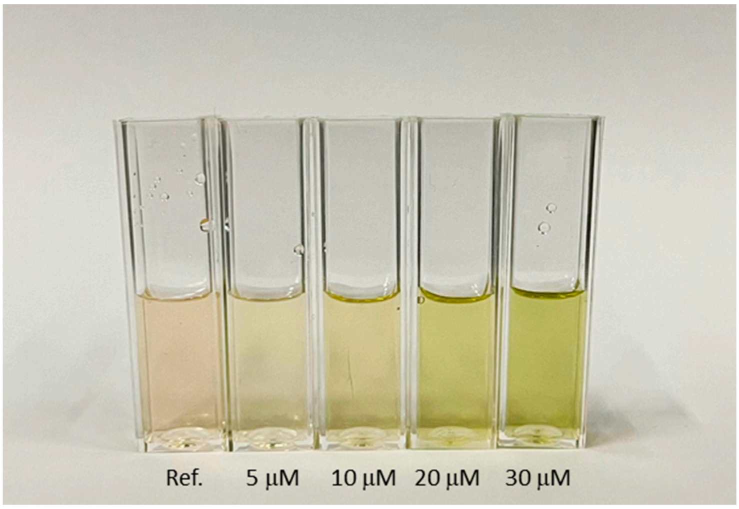

Figure S3. Color variations of the as-prepared NS-CDs sensing solution upon the addition of Cu(II) ions in the low range of concentrations.

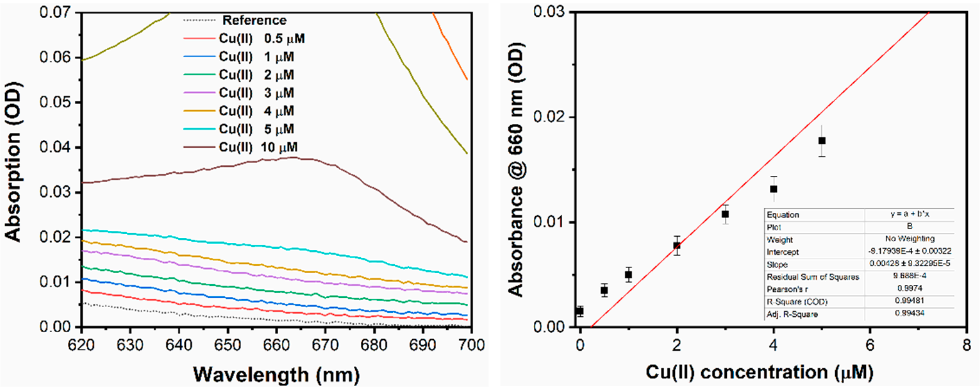

Figure S4. UV-vis absorption spectra (left) and calibration curve (right) of the as-prepared NS-CDs sensing solution upon the addition of Cu(II) ions in the low range of concentrations. The red line in the calibration curve represents the linear fit with the high-concentration data.

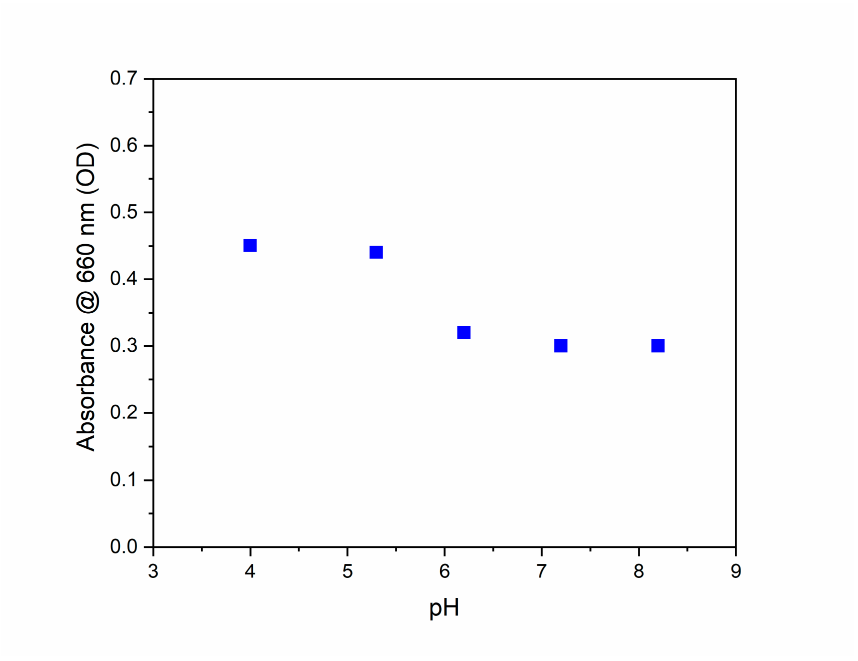

Figure S5. Dependance of absorbance at 660 nm of as-prepared NS-CDs sensing solution on the pH of the DI water samples with 100 μM of Cu(II).

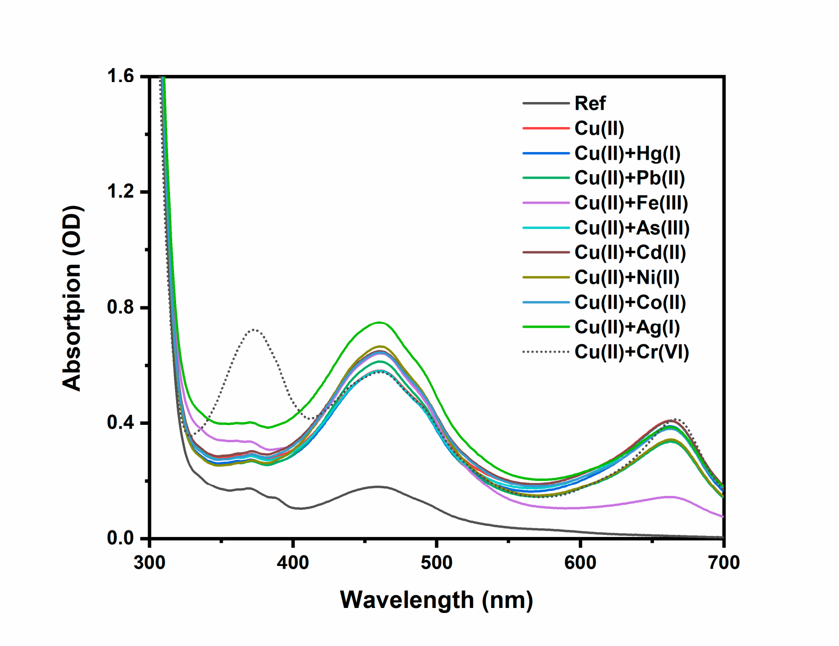

Figure S6. UV-vis absorption spectra of as-prepared NS-CDs sensing solution upon the addition of Cu(II) and other interfering HM ions at a concentration of 100 μM.

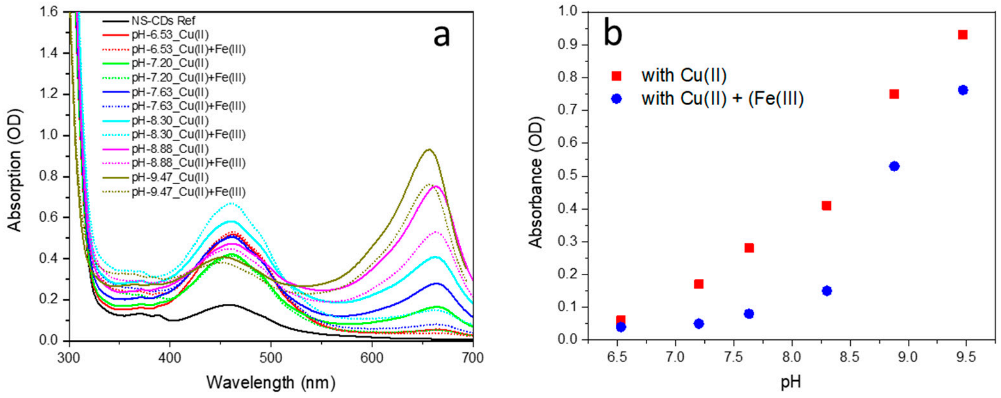

Figure S7. (a) UV-vis absorption spectra of NS-CDs sensing solution upon the addition of Cu(II) and Fe(III) at a concentration of 100 mM for different values of pH of sensing solution; (b) Absorbance at 660 nm with Cu(II) in the presence of Fe(III) (blue circles) and in absence (red squares) for different values of pH of sensing solution.

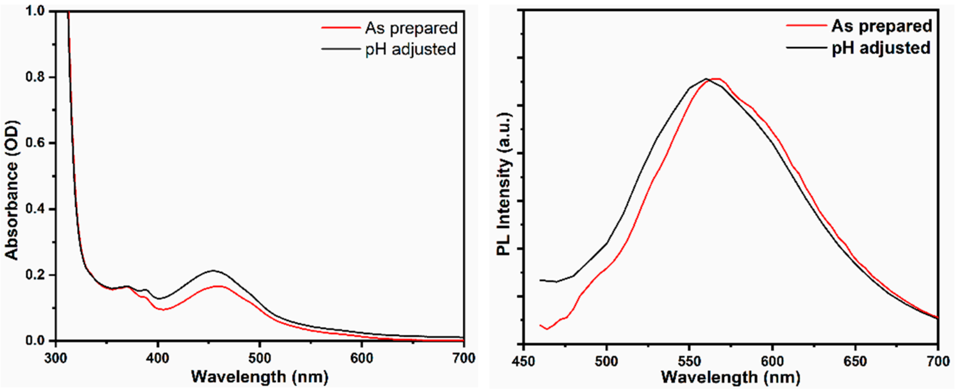

Figure S8. Comparison of UV-vis absorption spectra (left) and fluorescence spectra (right) of as-prepared NS-CDs sensing solution (red curves) and pH-adjusted sensing solution (black).

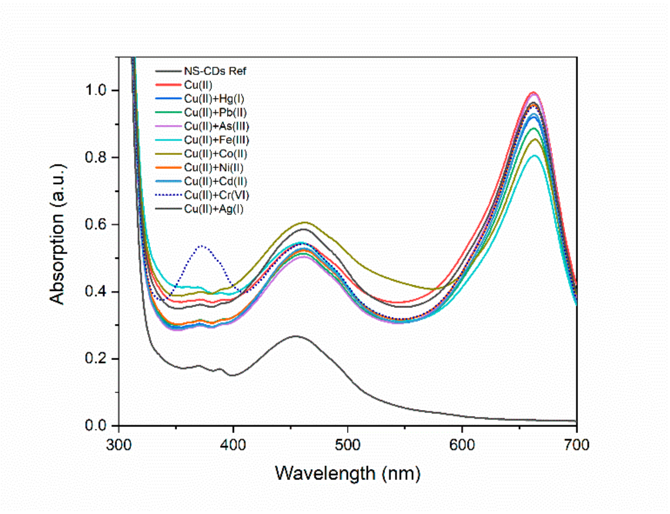

Figure S9. UV-vis absorption spectra of pH-optimized NS-CD sensing solution upon the addition of Cu(II) and other interfering HM ions at a concentration of 100 μM.

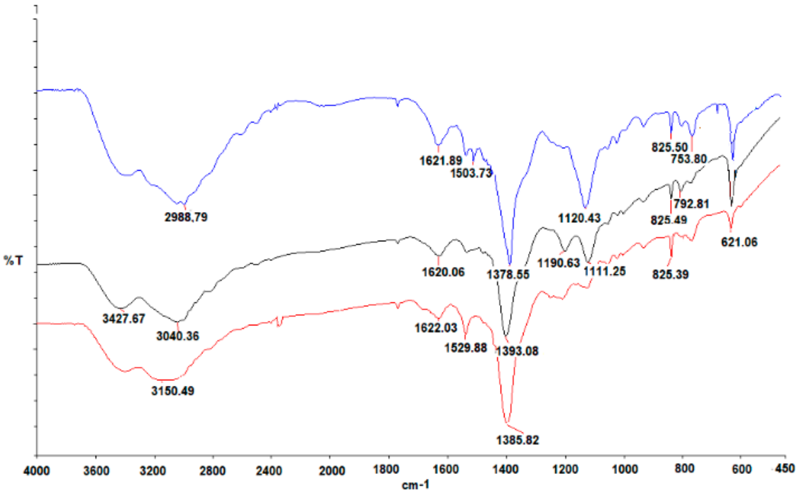

Figure S10. FTIR spectra of the dried samples: pH-optimized NS-CDs sensing solution (blue curve), NS-CDs + Cu(II) (black) and NS-CDs + Fe(III) (red).

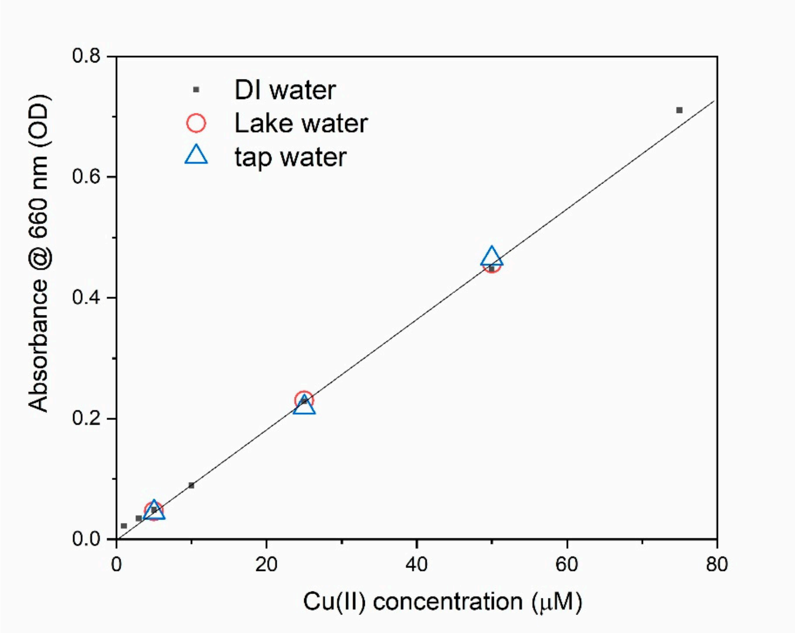

Figure S11. Measurements in real water samples in comparison with the calibration curve obtained in DI water.
